# Short-Term Feeding on Ecologically Distinct Dietary Plants Is Associated with Gut-Sample Bacterial and Archaeal Profiles in Adult *Anoplophora glabripennis*

**DOI:** 10.3390/insects17080756

**Published:** 2026-07-23

**Authors:** Hanshuo Liu, Ruohan Qi, Yi Tian, Lili Ren, Youqing Luo

**Affiliations:** 1 Beijing Key Laboratory for Forest Pest Control, Beijing Forestry University, Beijing 100083, China; lhansh@yeah.net (H.L.); qqqrh1999@bjfu.edu.cn (R.Q.); 2 Shijingshan District, Beijing Gardening and Greening Bureau, Beijing 100049, China; ty200609@sina.com

**Keywords:** *Anoplophora glabripennis*, adult feeding, gut microbiome, shotgun metagenomics, dietary plant category, dead-end trap plant

## Abstract

Ecological control studies of the Asian longhorned beetle (*Anoplophora glabripennis*) distinguish susceptible, resistant, and dead-end trap trees as functionally different plant categories. We used shotgun metagenomics to examine bacterial and archaeal profiles detected in adult gut samples after 72 h exposure to three dietary plants or prolonged water-only starvation. The study included 24 metagenomes, with three biological replicates per DietGroup × SexGroup combination. No time-zero gut samples were available, so the observed patterns remain superimposed on the beetles’ field history. The retained catalogue contained 152,895 bacterial genes and 9 archaeal genes. The original observed-richness difference was strongly correlated with host-depleted read depth and was not supported after common-depth rarefaction. Genus-level Bray–Curtis analysis detected a DietGroup × SexGroup interaction that persisted after depth adjustment and exclusion of low-yield samples. This interaction was exploratory because of the small within-cell sample size. Raw Bray–Curtis analysis of KEGG Orthology profiles showed a DietGroup association, but this association was not robust to direct-depth adjustment or Aitchison analysis. CAZy profiles were descriptive and showed no significant DietGroup effect. These results indicate short-term, depth-sensitive associations between dietary treatment and gut-sample bacterial and archaeal profiles. They do not establish resident status, microbial activity, or a physiological mechanism.

## Simple Summary

The Asian longhorned beetle is a destructive tree-boring insect that damages broad-leaved forests and shelterbelt systems. Adult beetles feed on twigs before reproduction, and different tree species may provide distinct nutritional and chemical environments. We examined whether 72 h feeding on susceptible, resistant, or dead-end trap dietary plants was associated with bacterial and archaeal profiles detected in adult gut samples. A fourth group underwent prolonged water-only starvation for about 120 h. We analyzed 24 individual gut metagenomes, with three beetles in each diet-by-sex combination. The original richness difference was not retained after common-depth rarefaction. An exploratory diet-by-sex association in genus composition persisted in sensitivity analyses, but the small sample size prevents a firm sex-specific conclusion. Predicted microbial gene profiles included carbohydrate, energy, transport, and plant-compound-related functions. These functional patterns were sensitive to sequencing depth and compositional treatment. The results generate hypotheses about short-term diet-associated gut-sample microbial variation, but they do not establish microbial activity or a mechanism of plant adaptation.

## 1. Introduction

The Asian longhorned beetle (ALB), *Anoplophora glabripennis* (Motschulsky) (Coleoptera: Cerambycidae), is a highly polyphagous wood-boring pest that damages broad-leaved forests and urban trees worldwide [1,2,3]. Adult ALBs conduct maturation or supplementary feeding on young shoots and leaves [4,5]. Females select oviposition sites on host stems, after which larvae develop within phloem and xylem [1,2].

In the ecological-control framework used for this experiment, dietary plants can be separated into three functional categories: susceptible plants, resistant plants, and dead-end trap plants [6,7]. The broader dead-end trap concept refers to plants that attract feeding or oviposition but reduce offspring survival [8]. Susceptible plants are readily used by ALBs and are expected to support feeding, oviposition, or offspring establishment; resistant plants reduce feeding or reproductive success through physical or chemical barriers; and dead-end trap plants attract adult feeding or oviposition but prevent successful larval development [6,7,9,10,11,12,13]. Accordingly, we selected Erbaiyang poplar (*Populus* × *xiaohei* var. *gansuensis*) as the susceptible dietary plant, Xinjiang poplar (*Populus alba* var. *pyramidalis*) as the resistant dietary plant, and Russian olive (*Elaeagnus angustifolia*) as the dead-end trap dietary plant. Russian olive is particularly relevant because adults can feed on shoots, whereas wound-induced gum secretion may encapsulate eggs or early larvae and thereby interrupt offspring survival [11,12,13]. Recent metabolomic evidence further showed that adult ALB feeding induces distinct metabolic defense responses in *E. angustifolia*, *P.* × *xiaohei* var. *gansuensis*, and *P. alba* var. *pyramidalis* [14]. This contrast provides an opportunity to test whether the adult gut microbiome is associated with dietary plants that are similar as immediate feeding substrates but distinct in ecological management function.

Microbes detected in insect guts can contribute to host nutrition, digestion, symbiosis, and interactions with chemically defended plants [15,16,17,18]. Microbes have been directly implicated in lignocellulose degradation in xylophagous insects [19,20], while broader insect–microbe literature supports roles in nutrient acquisition and processing of plant secondary compounds [15,16,17,18]. Studies of ALB larvae have revealed microbial and host-associated functions linked to lignocellulose degradation and nutritional ecology [21,22,23,24], and work on Anoplophora beetles has also shown that microbial communities differ across life stages and between larvae and adults [25,26]. Host adaptability of ALBs has, further, been linked to plant metabolomic characteristics and intestinal bacterial communities [27].

A 72 h feeding period was used as a short-term exposure window. The design did not aim to infer larval performance or long-term establishment in woody tissues. It tested whether gut-sample microbial taxa and gene profiles were associated with recent shoot exposure. Diet and environment can alter insect gut bacterial community structure [28], and diet-associated microbial responses can differ by sex in other host systems [29]. These studies provide comparative context but do not establish the same mechanism in adult ALBs. No gut samples were collected immediately after field collection or after the 48 h pretreatment. Consequently, treatment-associated patterns may also reflect residual host, field, and individual-history effects. Any short-term dietary association should therefore be interpreted as superimposed on this shared but unmeasured baseline.

Shotgun metagenomic sequencing allows simultaneous characterization of gut microbial taxonomic composition and functional gene potential [30]. For adult ALB feeding on fresh shoots, functional categories related to plant-derived carbohydrate utilization, carbohydrate-active enzymes, membrane transport, stress response, and candidate aromatic or terpenoid compound processing are particularly informative. Plant-associated microbial functions can contribute to processing or reducing defensive compounds in some herbivorous insects [31,32,33,34,35], while poplar and related woody plants contain diverse phenylpropanoid and other secondary metabolites that can shape herbivore interactions [36,37,38,39]. These considerations suggest that dietary plants differing in susceptibility, resistance, or dead-end trap function may impose distinct nutritional and chemical filters on the adult gut microbiome.

We used shotgun metagenomic sequencing to characterize bacterial and archaeal profiles detected in adult ALB gut samples under four short-term dietary conditions. These conditions comprised susceptible, resistant, and dead-end trap dietary plants plus prolonged water-only starvation. We tested associations with taxonomic composition, sex, and microbial functional gene profiles. We also summarized annotation-based genus–function links for selected pathways. Functional results represent gene abundance and inferred potential, not expression, enzyme activity, or evidence that microbes mediate plant resistance or the dead-end trap effect.

## 2. Materials and Methods

### 2.1. Insect Collection and Dietary Plant Materials

ALBs were collected from a roadside pure stand of *Acer negundo* in Zhugezhuang Town, Xiongxian County, Hebei Province, China (39.05298282° N, 116.05810328° E). A total of 103 adults were sexed, weighed, and maintained individually in transparent plastic boxes. Ninety-two beetles survived the 48 h water-only pretreatment. No time-zero gut samples were collected after field collection or after pretreatment. The pre-experimental microbiota may therefore have retained effects of *A. negundo*, field diet history, local exposure, and individual life history.

Three dietary plant species were used: Russian olive (*E. angustifolia*), Xinjiang poplar (*P. alba* var. *pyramidalis*), and Erbaiyang poplar (*P*. × *xiaohei* var. *gansuensis*). Seedlings of all three plant species were purchased from the Jiuquan-Jiayuguan region of Gansu Province, China. More than 100 seedlings were obtained for each plant species, with a basal stem diameter of approximately 15 mm. To ensure a stable and consistent food supply, hydroponic seedlings and branches from the same source were prepared. All seedlings were uniformly maintained in the greenhouse of the Plant Science Center of Beijing Forestry University and then transplanted into pots at the Forest Protection Station of Beijing Forestry University. Pots had an upper diameter × height of 26.5 cm × 27 cm and were filled with a vermiculite–peat mixed substrate. During the experiment, fresh semi-lignified current-year shoots bearing 3–4 leaves were collected continuously from each plant species and used as food for adult beetles.

### 2.2. Feeding Experiment and Experimental Design

Before starvation, beetles were provisionally grouped by sex and body mass. Because survival differed among provisional groups, the 92 survivors were reallocated after the 48 h pretreatment. Forty females and 40 males entered the 72 h treatment phase. Allocation was randomized within body-mass strata to provide 10 females and 10 males per treatment while minimizing differences in mean body mass. Beetles were reared individually at 25 ± 1 °C, 60% ± 5% relative humidity, and a 16 h light:8 h dark photoperiod. Feeding or refusal was monitored qualitatively, but consumed shoot mass, length, area, and tissue amount were not quantified. No beetle was excluded for refusal, visible injury, or other abnormal behavior.

All adults underwent a 48 h water-only starvation pretreatment. The three plant-fed groups then received fresh shoots for 72 h. The starvation group received distilled water but no plant food, resulting in approximately 120 h of water-only starvation. This group therefore represented prolonged food deprivation rather than a neutral no-plant control. Fresh shoots and water were renewed every 24 h, and all beetles were weighed at 0, 24, 48, and 72 h of treatment. Death was defined as complete cessation of activity, no appendage response, and no response after 30 s of continuous paintbrush stimulation. Severe weakness was defined as almost no movement after the same stimulation, with only slight antennal or limb movement. Four 72 h exclusions combined death and severe weakness because these states were not recorded separately immediately before dissection.

Fifty-seven beetles remained eligible at 72 h. Within each DietGroup × SexGroup subgroup, the mean 72 h body mass was calculated among eligible individuals. The three beetles closest to that subgroup mean were selected and immediately dissected before DNA extraction or DNA-quality assessment. This produced 24 sequenced gut samples, with three females and three males per treatment. The experimental allocation and metagenomic sequencing by treatment and sex are summarized in Table 1. Sample metadata are provided in Table S1, and participant flow, longitudinal body mass, selection, and qualitative feeding monitoring are reported in Table S14.

### 2.3. Gut Dissection and Sample Preservation

Before dissection, beetles were cold-anesthetized, and subsequent procedures were performed on ice. The surface was treated with 75% ethanol for 1 min and rinsed three times with sterile PBS. The entire gut was dissected using sterile forceps after removal of attached tracheae and Malpighian tubules. The gut surface was gently rinsed with sterile PBS. Luminal contents were retained, so each sample comprised gut tissue and its contents. This design cannot distinguish resident microbes from recently ingested or transient plant-associated microbes.

Gut samples were immediately frozen in liquid nitrogen and then stored at −80 °C until DNA extraction and sequencing. Metagenomic sequencing was performed by Majorbio Bio-Pharm Technology Co., Ltd. (Shanghai, China).

### 2.4. DNA Extraction, Library Construction, and Metagenomic Sequencing

Total DNA was extracted from gut samples using the E.Z.N.A.^®^ Soil DNA Kit (Omega Bio-tek, Inc., Norcross, GA, USA) according to the manufacturer’s instructions. DNA integrity was assessed using 1% agarose gel electrophoresis, and DNA concentration and purity were measured. The extracted DNA was then randomly fragmented using a Covaris M220 instrument (Covaris, Woburn, MA, USA), and fragments of approximately 350 bp were selected for library construction.

Paired-end sequencing libraries were constructed using the NEXTFLEX^®^ Rapid DNA-Seq Kit (Bioo Scientific Corporation, Austin, TX, USA). The main steps included adapter ligation, bead-based size selection to remove self-ligated adapter fragments, PCR amplification to enrich library templates, and purification of PCR products. The constructed libraries were sequenced on the Illumina NovaSeq™ X Plus platform (Illumina, Inc., San Diego, CA, USA).

### 2.5. Quality Control, Assembly, Gene Prediction, and Non-Redundant Gene Catalog Construction

Adapters and low-quality reads were removed with fastp v0.20.0 using -q 20, -l 50, -n 0, --detect_adapter_for_pe, --cut_front, --cut_tail, --cut_window_size 10, and --cut_mean_quality 20 [40]. Only paired clean reads were retained for downstream analyses. Host reads were identified with BWA-MEM v0.7.17 against the *A. glabripennis* assembly GCA_000390285.1 (Agla_1.0) [41]. Default alignment settings and no MAPQ threshold were used. If either mate aligned, including a secondary or supplementary alignment, the complete pair was removed.

Host-depleted reads were assembled separately for each sample with MEGAHIT v1.2.9 [42]. Parameters were --k-min 47, --k-max 97, --k-step 10, --min-contig-len 300, --mem-flag 1, and -t 20. Prodigal v2.6.3 was run in metagenomic mode (-p meta), and predicted genes shorter than 100 bp were removed [43]. Partial genes were retained. CD-HIT/CD-HIT-2D v4.6.1 was used to construct the non-redundant gene catalogue with 90% sequence identity and 90% coverage relative to the shorter sequence (-c 0.90 and -aS 0.90) [44]. SOAPaligner v2.21 mapped paired reads to the catalogue using a 300–500 bp insert range, -r 1, -M 4, -v 20, and -c 0.95 [45]. One best location was chosen randomly for tied mappings, and unpaired reads were excluded. Gene abundances were normalized as TPM. Complete parameters are provided in Table S16.

### 2.6. Taxonomic and Functional Annotation

NR taxonomic alignment used DIAMOND v2.0.13 and the September 2022 NR release [46]. Each gene retained up to 10 candidate hits at E ≤ 1 × 10^−5^; no additional identity, query-coverage, or subject-coverage threshold was applied. The company lowest-common-ancestor workflow then assigned the lowest taxonomic rank shared by more than 50% of candidate hits. Rank-level failures were unclassified, whereas ambiguous or inconsistent database assignments were retained as such. The raw LCA output contained 979 records; the retained table contained 585 records after excluding Plant, Chloroplast, Mitochondria, Fungi, and other non-target eukaryotic annotations and retaining only d__Bacteria or d__Archaea.

COG 2020 and KEGG September 2022 annotations used DIAMOND with an E-value threshold of 1 × 10^−5^ and the highest-scoring hit [47,48]. CAZy v8 annotation used HMMER v3.1b2 hmmscan against the dbCAN2 HMM collection [49,50,51]. Functional abundance was calculated only from genes retained after bacterial/archaeal filtering.

### 2.7. Statistical Analysis

Primary statistical analyses were performed in R 4.1.3 with the tidyverse, vegan, ggtext, pheatmap, and readxl packages. Reviewer-requested sensitivity analyses were conducted in Python 3.12 with NumPy 2.3.5 and pandas 3.0.1, and PERMANOVA results were independently validated in R 4.5.2 with vegan 2.7-5 and permute 0.9-10. Grouping variables were DietGroup, SexGroup, their combined eight-level Group, FeedingStatus, and PlantDietOnly. DietGroup comprised E, S, X, and J, and SexGroup comprised female and male beetles.

Alpha diversity was calculated from the bacterial/archaeal genus count matrix. Indices included observed richness (sobs), Shannon diversity, Simpson diversity, and Pielou evenness. The original non-rarefied comparisons used Kruskal–Wallis or Wilcoxon tests with Benjamini–Hochberg correction where applicable. Depth sensitivity was evaluated by Spearman correlation with host-depleted reads and retained assigned reads. Observed richness was also rarefied to the minimum bacterial/archaeal genus depth of 120 reads over 1000 iterations. Permutation *p* values used 4999 permutations and seed 20,260,717. Except for alpha diversity, main abundance analyses used TPM values.

Beta diversity used Bray–Curtis distances and principal coordinates analysis. Primary PERMANOVA models were fitted with adonis2() as DietGroup + SexGroup + DietGroup:SexGroup [52]. PERMDISP used betadisper() and permutest() [53]. Sensitivity analyses used 9999 permutations and included direct assigned-read depth or host-depleted depth as covariates. They also excluded samples with fewer than 1000 retained genus-assigned reads. Functional sensitivity checks applied per-sample closure and CLR/Aitchison distances after zero replacement. All interaction and sex-stratified results were considered exploratory because each DietGroup × SexGroup cell contained three samples.

Global taxon and pathway screening used Kruskal–Wallis tests, with Benjamini–Hochberg correction across the tested feature set. The full KEGG Level 3 table was merged by pathway name and filtered to pathways detected in at least three samples. All 307 retained pathways were tested before any focused interpretation. The displayed 18 pathways were selected post hoc from candidates with BH-FDR < 0.10 but not BH-FDR < 0.05, based on biological interpretability. They were treated as descriptive candidates rather than an a priori confirmatory set.

Sex-stratified single-genus tests and LEfSe were exploratory screens. LEfSe used DietGroup as the class variable, LDA score > 2.0, and nominal *p* < 0.05 without multiple-testing correction [54]. Candidate taxa were not treated as confirmed biomarkers because of *n* = 3 per DietGroup × SexGroup cell.

Annotation-based genus–function summaries linked NR/LCA genus and KEGG Level 3 annotations through the same retained bacterial/archaeal gene record. Gene TPM values were summed by genus, pathway, and sample, then averaged within DietGroup. The displayed taxa were the most abundant genus annotations, with unresolved inconsistent assignments retained for transparency. The analysis was implemented with a Perl workflow company. This is not a correlation, MAG, binning, expression, or activity analysis. Contribution values therefore represent inferred annotation-based abundance links and may reflect database representation.

## 3. Results

### 3.1. Adult Gut Metagenomes Yielded a Filtered Microbial Gene Catalogue for Comparative Analysis

A total of 24 adult ALB gut metagenomes were analyzed. Raw Q20 values ranged from 92.53% to 94.13%, and raw Q30 values ranged from 87.82% to 90.18%. GC content ranged from 34.62% to 39.70%. Clean reads accounted for 96.47–97.35% of raw reads, and clean bases accounted for 91.99–94.69% of raw bases (Table S2).

Host-depleted reads accounted for 0.56–51.78% of raw reads, showing substantial variation in non-host sequence recovery (Table S2). Three samples had fewer than 1000 reads assigned to retained genus features. Observed richness was strongly correlated with host-depleted read count (Spearman ρ = 0.904, permutation *p* = 0.0002; Table S15). Assembly and gene prediction produced 487,969 genes before dereplication. CD-HIT generated 193,012 non-redundant genes with a total length of 108,859,473 bp (Tables S3 and S4A).

The complete NR/LCA table contained 979 annotation records, of which 585 bacterial or archaeal records were retained (Table S4B,C). The filtered catalogue contained 152,904 genes, including 152,895 bacterial and 9 archaeal genes. Its total length was 93,309,957 bp, and the mean gene length was 610.25 bp (Table S4A). No fungal genes were included in the main taxonomic or functional analyses.

### 3.2. Bacterial and Archaeal Profiles Detected in Gut Samples Varied Descriptively Among Treatments

**Figure 1:**
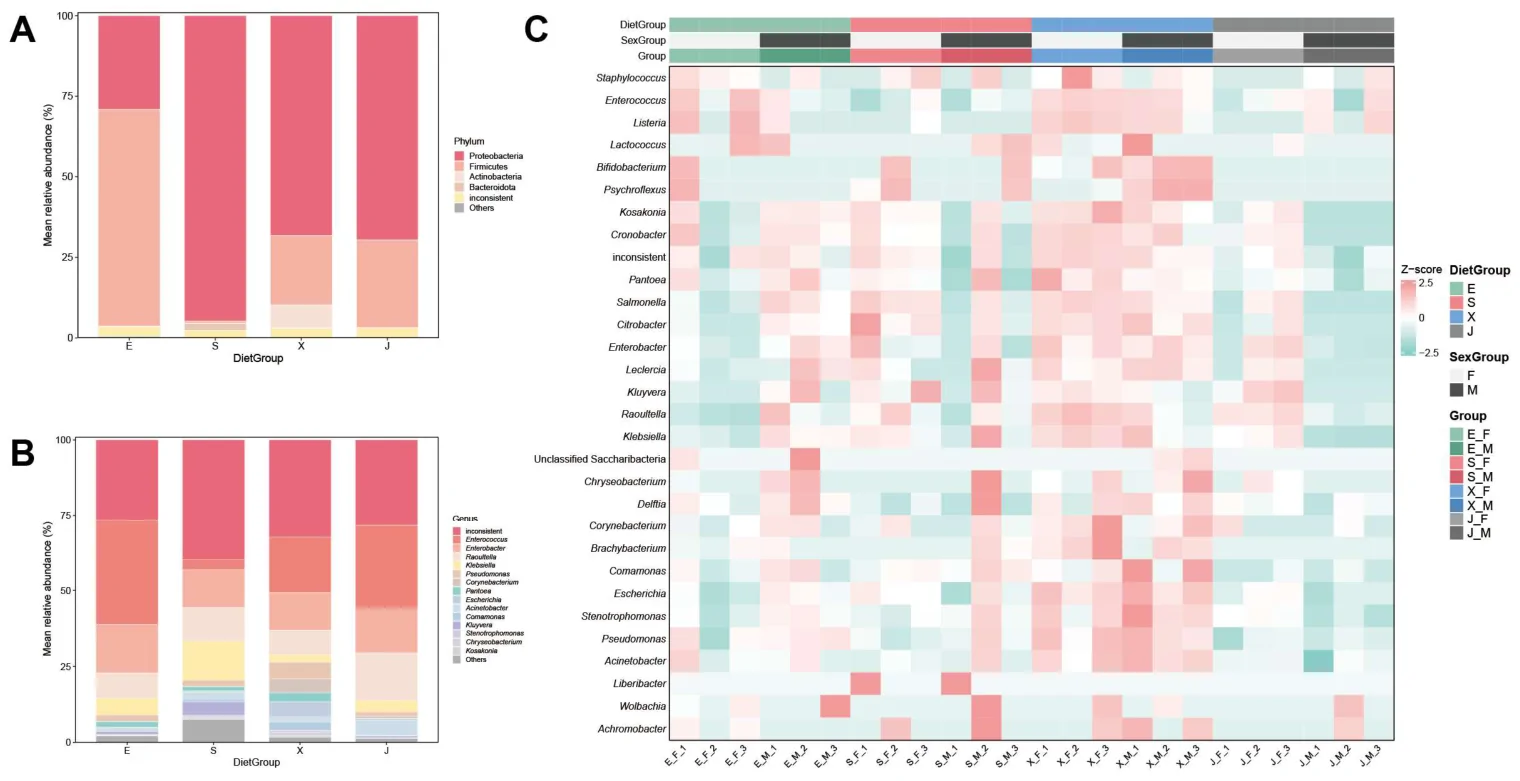
Bacterial and archaeal taxonomic profiles detected in adult *A. glabripennis* gut samples. (**A**) Mean relative abundance of major phyla. (**B**) Mean relative abundance of the top 15 genera. (**C**) Row-wise Z-score heatmap of the top 30 genera across samples. Panels are displayed at enlarged scale for readability. Low-abundance taxa were grouped as “Others”, and ambiguous assignments were retained as “inconsistent”. The profiles do not distinguish resident gut microbes from plant-associated, transient, or reagent-derived sources.

Taxonomic profiles were summarized from the filtered bacterial/archaeal NR/LCA abundance matrix (Table S4C). Proteobacteria and Firmicutes dominated, with Actinobacteria and Bacteroidota at lower relative abundance (Figure 1A). The relative proportions varied among E, S, X, and J samples. These patterns are descriptive because plant-associated and reagent controls were unavailable.

Dominant annotated genera included *Enterococcus*, *Enterobacter*, *Raoultella*, *Klebsiella*, *Pseudomonas*, *Corynebacterium*, *Pantoea*, *Escherichia*, *Acinetobacter*, *Comamonas*, *Kluyvera*, *Stenotrophomonas*, *Chryseobacterium*, and *Kosakonia* (Figure 1B). Sample-level profiles varied within and among treatments (Figure 1C). These genera are described as detected in gut samples. Their resident status cannot be established without plant-surface, endophyte, reagent, and gut-fraction controls.

Ten named genera met the study’s core-detection rule, and 17 additional genera met the prevalent rule (Figure S1; Table S5A,B). The named core set included *Enterococcus*, *Enterobacter*, *Raoultella*, *Klebsiella*, *Pseudomonas*, *Acinetobacter*, *Pantoea*, *Escherichia*, *Stenotrophomonas*, and *Delftia*. Here, core and prevalent denote repeated detection in these gut samples, not proof of stable residence.

**Figure 2:**
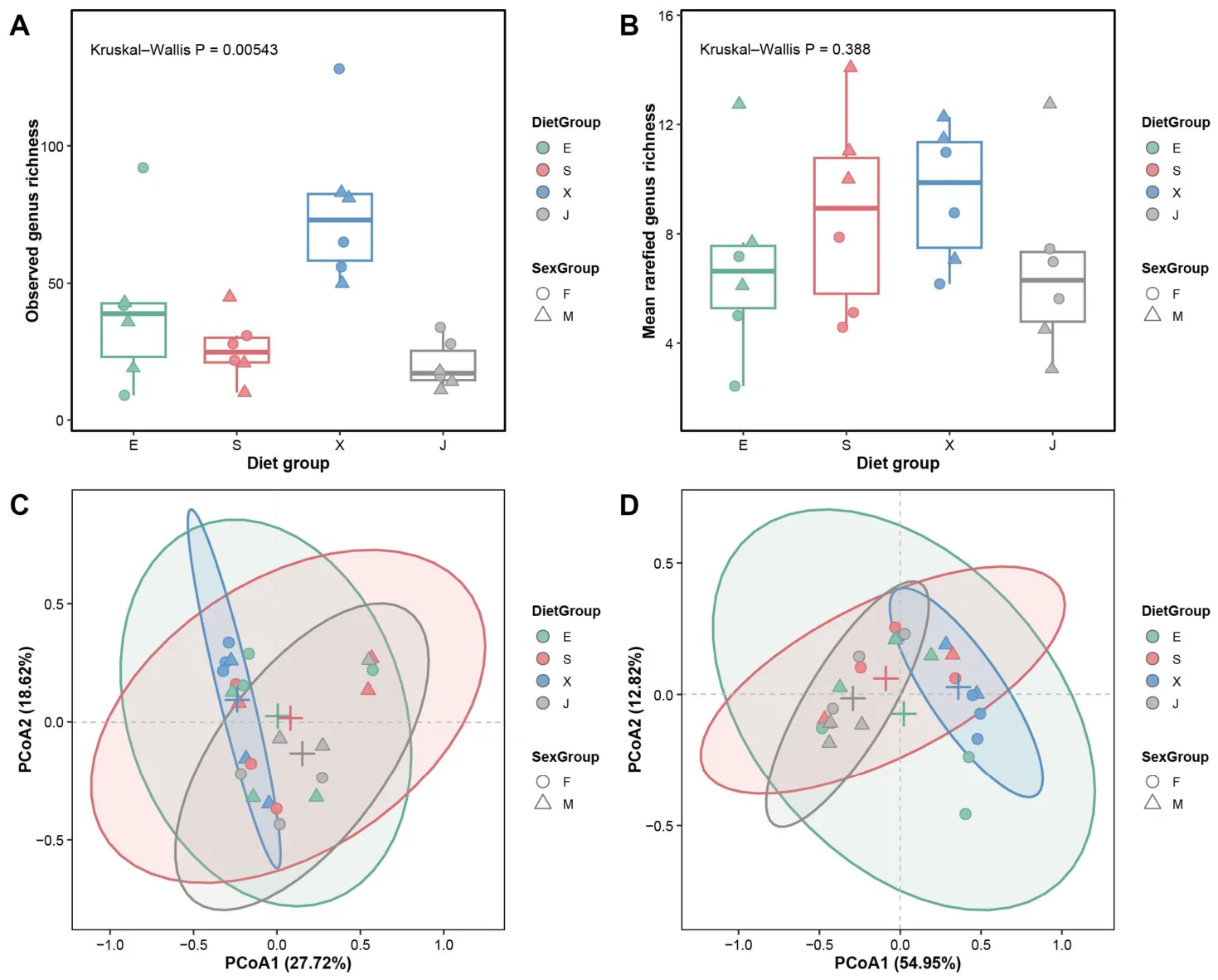
Richness sensitivity and beta-diversity profiles. (**A**) Original non-rarefied observed genus richness (Kruskal–Wallis *p* = 0.00543). (**B**) Mean observed genus richness after 1000 rarefactions to 120 retained bacterial/archaeal genus reads (permutation *p* = 0.3878). (**C**) Genus-level Bray–Curtis PCoA with 95% t-distribution ellipses. (**D**) KEGG KO Bray–Curtis PCoA with 68% normal-theory ellipses. Point shapes denote sex. Colored plus signs indicate DietGroup centroids. The ellipses are visual guides and do not represent independent evidence of separation. Inference was based on PERMANOVA, PERMDISP, and the sensitivity analyses in Table S15.

In the original non-rarefied analysis, observed richness differed among DietGroups (Kruskal–Wallis H = 12.6605, *p* = 0.00543; Figure 2A; Table S6). The X group had the largest raw mean sobs value. However, sobs was strongly associated with host-depleted read depth. After rarefaction to 120 retained bacterial/archaeal genus reads, the DietGroup difference was not significant (permutation *p* = 0.3878; Figure 2B; Table S15). The apparent X-group richness increase was therefore not considered robust.

Shannon diversity, Simpson diversity, and Pielou evenness did not differ among DietGroups (*p* = 0.242, 0.200, and 0.883, respectively; Figure S2; Table S6). Together with the rarefaction result, these analyses do not support a depth-independent treatment effect on alpha diversity.

### 3.3. Genus Composition Showed an Exploratory DietGroup × SexGroup Association

Genus composition was evaluated with Bray–Curtis PCoA using the retained bacterial/archaeal genus TPM table. PCoA1 and PCoA2 explained 27.72% and 18.62% of variation (Figure 2C). The ordination is a visual summary; inferential interpretation was based on PERMANOVA and PERMDISP.

The original factorial PERMANOVA detected a DietGroup × SexGroup interaction (R^2^ = 0.2020, F = 1.8345, *p* = 0.0178; Table S7). A 9999-permutation reanalysis gave *p* = 0.0142 (Table S15). The interaction persisted after adjustment for retained assigned-read depth (*p* = 0.0023) and after excluding three low-yield samples (*p* = 0.0073). DietGroup and SexGroup main terms were not significant in the original model. Because each cell contained three samples, the interaction was treated as an exploratory association rather than a confirmed sex-specific response.

PERMDISP did not detect significant dispersion differences among DietGroups, SexGroups, or the eight combined groups (Table S7). This reduced concern that the original interaction arose only from dispersion heterogeneity. It did not resolve uncertainty caused by small cell size, depth variation, or field-derived baseline differences.

In sex-stratified exploratory analyses, DietGroup explained genus composition in females (R^2^ = 0.4464, *p* = 0.0050) but not males (R^2^ = 0.3275, *p* = 0.1822; Table S7). Neither sex-stratified PERMDISP test was significant. These results describe the current dataset and should not be interpreted as a physiological mechanism or a population-level female-specific effect.

Four genera showed nominal DietGroup associations in females, and one did so in males (Figure S3; Table S8). None remained significant after Benjamini–Hochberg correction. They were therefore retained only as exploratory candidates.

Sex-stratified LEfSe also identified candidate taxa using nominal *p* < 0.05 and LDA score > 2.0 (Table S9). These candidates were not FDR-controlled and were not treated as validated biomarkers.

### 3.4. KEGG Profiles Showed a Depth- and Composition-Sensitive DietGroup Association

After prevalence filtering, 4942 KEGG Orthology features were retained from the bacterial/archaeal gene set. Bray–Curtis PCoA1 and PCoA2 explained 54.95% and 12.82% of functional-profile variation (Figure 2D). The ordination was used as a visual summary; overviews of the top 30 KOs and selected KEGG Level 2 categories are provided in Figures S4 and S5, respectively.

The original raw-TPM Bray–Curtis PERMANOVA detected a DietGroup term (R^2^ = 0.3018, F = 3.1092, *p* = 0.003; Table S10A). A 9999-permutation reanalysis produced *p* = 0.0075, and per-sample closure produced *p* = 0.0140 (Table S15). However, the DietGroup term was not significant after direct assigned-read depth was added (*p* = 0.2320) or under Aitchison distance (*p* = 0.3122). The KEGG DietGroup association was therefore considered exploratory and method-sensitive.

PERMDISP did not detect a significant DietGroup dispersion difference in the original KO analysis (*p* = 0.113; Table S10). This result addresses dispersion but not the strong relationship between assigned-read depth and recovered KO features. We therefore do not conclude that functional profiles were primarily determined by diet.

### 3.5. Post Hoc Functional Profiles Identified Candidate Plant-Substrate-Related Pathways

**Figure 3:**
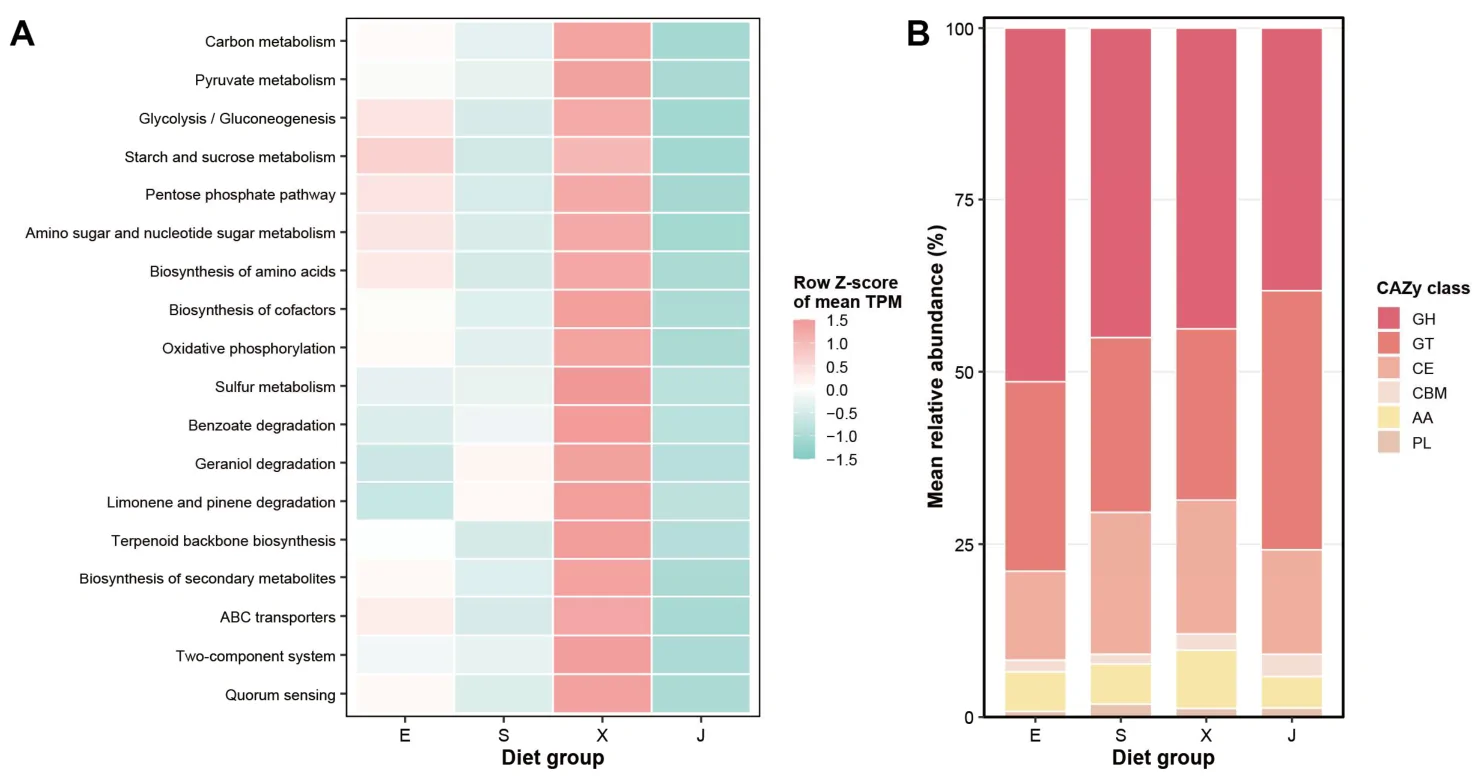
Descriptive functional annotation profiles in the retained bacterial/archaeal gene set. (**A**) DietGroup mean abundance of 18 post hoc KEGG Level 3 candidates, displayed as row-wise Z-scores of mean TPM. Pathways were selected after the complete prevalence-filtered screen from candidates with BH-FDR < 0.10 but not BH-FDR < 0.05. (**B**) Mean relative abundance of CAZy classes by DietGroup. Neither panel indicates confirmatory pathway differences, gene expression, enzyme activity, or metabolic activity.

The complete KEGG Level 3 screen retained 307 pathways detected in at least three samples. In the original raw-TPM screen, no pathway met BH-FDR < 0.05, whereas 239 met BH-FDR < 0.10 (Table S15). For the full 307-pathway matrix, the closed Bray–Curtis DietGroup term was significant before depth adjustment (R^2^ = 0.4516, *p* = 0.0097) but not after assigned-read-depth adjustment (*p* = 0.1420) or with Aitchison distance (*p* = 0.2864; Table S15). PERMDISP also detected DietGroup dispersion differences after closure and under Aitchison distance (*p* = 0.0365 and 0.0295, respectively). The displayed 18 pathways were selected after inspection from the FDR < 0.10 candidates because they were biologically interpretable. They were not prespecified and were not treated as confirmatory discoveries (Figure 3A; Table S11).

For visualization, pathway abundance was calculated as the mean TPM within each DietGroup. The X group showed larger mean TPM values and the J group smaller values across the displayed pathways (Table S11). The panel included carbohydrate, central carbon, energy, biosynthetic, transport, aromatic-compound, and terpenoid-related annotations. Because selection was post hoc and the profiles were depth- and composition-sensitive, these patterns are descriptive candidates (Figure 3A).

CAZy profiles were evaluated descriptively. Sample S_M_1 had an all-zero CAZy profile because no genes retained after bacterial/archaeal filtering had CAZy annotations. The sample was retained in other analyses because its bacterial/archaeal catalogue contained 8040 assigned reads and 125 nonzero genes. The DietGroup term was not significant either when S_M_1 was included as an all-zero numerical row (R^2^ = 0.1682, *p* = 0.0770) or when it was excluded from the composition-defined analysis (R^2^ = 0.1762, *p* = 0.0926; Table S12). Thus, its analysis-specific exclusion did not change the statistical conclusion. The DietGroup-level CAZy class profile is shown descriptively in Figure 3B; supplementary class- and top-20-family composition views are provided in Figure S6A and S6B, respectively, and the detailed plant-cell-wall-related family heatmap remains in Figure S8. Detected GH, CE, AA, CBM, and PL families indicate annotated gene repertoire, not diet-driven activity.

### 3.6. Inferred Genus-Associated Functional Annotation Patterns Were Uneven

**Figure 4:**
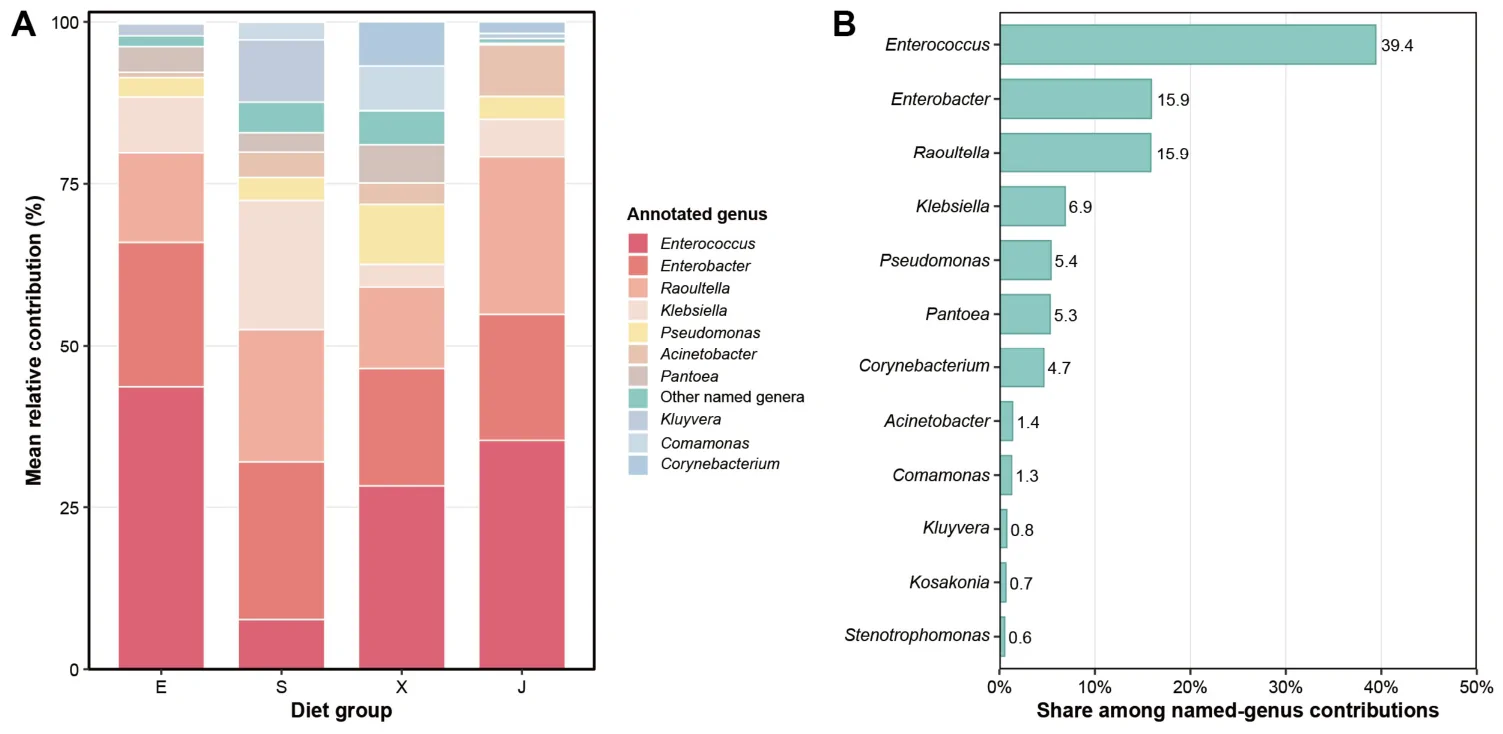
Annotation-based genus contributions to the 18 displayed KEGG Level 3 pathways. (**A**) Mean relative contribution composition of major named genus annotations across DietGroups; low-contribution named annotations were grouped as other named genera. (**B**) Total contribution share of the 12 leading named genera. Values were inferred by linking taxonomic and functional annotations through shared bacterial/archaeal gene records. They do not demonstrate gene expression, enzyme activity, correlation, or a genome-resolved functional role.

The annotation-based contribution table linked the 18 displayed pathways to 28 genus-level annotations through shared retained genes (Table S13A). Prominent named annotations included *Enterococcus*, *Enterobacter*, *Raoultella*, *Klebsiella*, *Pseudomonas*, *Pantoea*, and *Corynebacterium*. *Enterococcus* had the largest summed annotation-based contribution among named genera (Figure 4B and Figure S7; Table S13B).

Mean inferred contribution composition differed descriptively among DietGroups (Figure 4A; Table S13C). *Enterococcus* was prominent in E, X, and J, whereas *Enterobacter*, *Raoultella*, and *Klebsiella* had larger relative shares in S. These summaries depend on gene annotation and database representation.

The detailed Genus × KEGG Level 3 matrix is provided in Figure S9 and Table S13D. It shows annotation patterns, not direct functional activity by a genus. Validation would require genome-resolved binning, contig-level taxonomy, expression data, or isolate-based functional tests.

## 4. Discussion

### 4.1. Short-Term Dietary Treatments Were Associated with Variation in Adult Gut-Sample Bacterial and Archaeal Profiles

Short-term exposure to ecologically distinct dietary treatments was associated with variation in bacterial and archaeal profiles detected in adult ALB gut samples. The evidence is descriptive and hypothesis-generating. It is bounded by the lack of a time-zero baseline, prolonged starvation in J, variable microbial read recovery, and three samples per diet-by-sex cell.

The dominant bacterial phyla and genera overlap with taxa reported from Anoplophora and other wood-feeding beetles [21,24,25,26]. Prior studies document host resistance, metabolic defense responses, and secondary-metabolite diversity among ALB hosts and related woody plants [9,10,14,36,37,38,39]. Because plant chemistry and consumed plant amount were not quantified here, the specific dietary drivers of the observed profiles cannot be identified.

The J group cannot be interpreted as a neutral absence-of-plant control. It experienced approximately 120 h of water-only starvation, whereas plant-fed beetles received shoots after the shared 48 h pretreatment. Differences involving J may reflect food absence, starvation stress, gut emptying, microbial load, peristalsis, immunity, or metabolism.

### 4.2. Exploratory Taxonomic Associations Involved Diet and Sex, Whereas KEGG Patterns Were Method-Sensitive

The genus-level DietGroup × SexGroup interaction was reproducible in Bray–Curtis sensitivity analyses and persisted after depth adjustment and low-yield exclusion (Tables S7 and S15). The sex-stratified female association was present in this dataset, but each DietGroup × SexGroup cell contained three samples. The result is therefore an exploratory association requiring larger, independently replicated experiments.

Feeding amount, reproductive status, ovarian development, fecundity, and hormone levels were not measured. Body mass was used during allocation and sequencing selection, which may also limit generalization. Physiological explanations for the observed sex-related pattern would therefore be speculative and are not advanced here.

The original KEGG Bray–Curtis analysis showed a DietGroup association, but the result was sensitive to assigned-read depth and Aitchison transformation. Functional redundancy describes the maintenance of overlapping community functions despite taxonomic variation or disturbance [55,56]. Although this concept provides a possible framework for comparing taxonomic and functional patterns, the depth-sensitive KEGG results do not demonstrate it here. They identify method-sensitive gene-profile patterns for future validation.

### 4.3. Functional Annotations Provided Descriptive Candidate Patterns Related to Plant-Derived Substrates

The 18 displayed KEGG Level 3 pathways were selected post hoc from the complete screened set. No pathway met BH-FDR < 0.05 in the original raw-TPM screen. The displayed carbohydrate, energy, biosynthetic, transport, aromatic, and terpenoid annotations should therefore be interpreted as candidate patterns.

Several displayed pathways are compatible with microbial capacity to process plant-derived substrates. Plant-cell-wall-degrading genes have been reported in beetles and their associated microbiomes, while broader reviews summarize lignocellulose-degradation mechanisms across taxa [19,20,21,22,23,24,57,58,59]. In this study, pathway abundance was not robust to every depth or compositional treatment, so substrate processing remains a hypothesis.

CAZy profiles showed no significant DietGroup effect and were retained as a descriptive inventory. The detected GH, CE, AA, CBM, and PL annotations indicate gene repertoire only. Cellulase activity has previously been examined in adult ALBs in relation to host use [60]. However, enzyme activity and its host or microbial origin cannot be inferred from the bacterial/archaeal CAZy gene inventory reported here. Sample S_M_1 lacked retained bacterial/archaeal CAZy annotations.

Aromatic- and terpenoid-related annotations may be relevant to dietary plant chemistry, but they do not measure plant metabolites or detoxification. Microbial processing of plant defenses has been demonstrated in other insect systems [31,32,33,34,35]. Here, validation would require plant metabolomics, metatranscriptomics, isolate assays, enzyme measurements, and host-performance tests.

### 4.4. Management-Relevant Dietary Treatments Were Associated with Gut-Sample Microbial Variation

The selected plants represent distinct roles in ALB management [6,7,9,10,11,12,13,14]. However, these ecological labels do not make the adult feeding substrates experimentally equivalent. The dead-end trap effect of *E. angustifolia* is understood mainly through oviposition and offspring mortality, not adult gut microbial function [11,12,13].

This study does not show that gut microbes mediate the dead-end trap effect, overcome plant defenses, or determine beetle performance. It shows only that gut-sample bacterial, archaeal, and gene profiles were associated with short-term treatment under the present design.

Genus–function summaries identify annotation-linked taxa and pathways for future work (Figure 4 and Figure S9; Table S13). Database representation can influence these links. Genome-resolved and experimentally validated analyses are required before assigning a functional role to any genus.

### 4.5. Limitations and Future Directions

The study lacked gut samples at field collection or after the 48 h pretreatment. Treatment-associated patterns therefore remain superimposed on the original *A. negundo* host, field environment, and individual history. Plant-surface, shoot-endophyte, reagent-negative, and gut-fraction controls were also unavailable. Because luminal contents were retained, detected microbes cannot be classified confidently as resident rather than transient, dietary, or processing-derived.

The starvation treatment involved approximately 120 h of water-only deprivation and was not a neutral control. Feeding was monitored qualitatively, but ingestion amount was not measured. Body mass was recorded, and individuals closest to each 72 h DietGroup × SexGroup mean were selected. Reproductive status was not recorded. These factors limit physiological interpretation of treatment and sex associations.

Host-depleted and assigned-read depths varied strongly, and observed richness was not robust after common-depth rarefaction. KEGG DietGroup results were sensitive to depth adjustment and compositional analysis. The sample size was three per DietGroup × SexGroup cell. Consequently, richness, LEfSe, single-genus, sex-stratified, pathway, and contribution results are exploratory. Larger studies with matched controls, quantitative feeding, plant chemistry, genome-resolved analyses, and functional validation are needed.

## 5. Conclusions

Short-term dietary treatment was associated with exploratory variation in bacterial and archaeal profiles detected in adult *A. glabripennis* gut samples. The original richness difference was not supported after common-depth rarefaction. A genus-level DietGroup × SexGroup interaction persisted in Bray–Curtis sensitivity analyses, but *n* = 3 per cell precludes a firm sex-specific conclusion. Raw KEGG Bray–Curtis profiles showed a DietGroup association that was sensitive to depth adjustment and Aitchison analysis. CAZy and genus–function results were descriptive and annotation-based.

These findings do not identify resident microbes, active metabolic functions, or mechanisms of plant resistance and dead-end trapping. They provide a bounded, hypothesis-generating dataset for future studies that include time-zero, plant, reagent, and gut-fraction controls; quantitative feeding; larger sample sizes; and functional validation.

## Figures and Tables

**Table 1:** Experimental allocation, 72 h eligibility, exclusions, and metagenomic sequencing by dietary treatment and sex.

DietGroup	Ecological Category	Treatment	Entered Treatment	Eligible at 72 h	Excluded During Treatment	Sequenced
E	Susceptible dietary plant	Erbaiyang poplar	10 F/10 M	9 F/8 M	1 F/2 M	3 F/3 M
S	Dead-end trap dietary plant	Russian olive	10 F/10 M	8 F/8 M	2 F/2 M	3 F/3 M
X	Resistant dietary plant	Xinjiang poplar	10 F/10 M	8 F/7 M	2 F/3 M	3 F/3 M
J	Prolonged water-only starvation	No plant food	10 F/10 M	5 F/4 M	5 F/6 M	3 F/3 M

## Data Availability

Raw metagenomic reads and sample metadata are publicly available in the NCBI Sequence Read Archive under BioProject PRJNA1480437 (Diet plant-associated gut metagenomes of adult *Anoplophora glabripennis*; released 22 June 2026). The original contributions presented in this study are included in the article/Supplementary Materials. Further inquiries can be directed to the corresponding author.

## Supplementary Materials

The following supporting information can be downloaded at: https://www.mdpi.com/article/10.3390/insects17080756/s1, Figure S1: Prevalence and relative abundance of genus-level taxa; Figure S2: Alpha diversity indices; Figure S3: Exploratory female-only genus differences; Figure S4: Top 30 KEGG Orthology categories; Figure S5: Selected KEGG Level 2 categories; Figure S6: CAZy class and top-family composition; Figure S7: Total inferred genus contribution; Figure S8: Descriptive plant-cell-wall-related CAZy family heatmap; Figure S9: Detailed Genus × KEGG Level 3 annotation-based contribution heatmap; Table S1: Sample metadata; Table S2: Sequencing and host-depletion summary; Table S3: Assembly and ORF statistics; Table S4: Non-redundant catalogue and filtered bacterial/archaeal dataset; Table S5: Core and prevalent genera; Table S6: Alpha diversity; Table S7: Genus-level PERMANOVA and PERMDISP; Table S8: Sex-stratified genus screening; Table S9: Exploratory LEfSe results; Table S10: KEGG KO PERMANOVA and PERMDISP; Table S11: Post hoc KEGG Level 3 profiles; Table S12: CAZy profiles and S_M_1 inclusion/exclusion sensitivity; Table S13: Annotation-based genus–function summaries; Table S14: Sample flow, body mass, and feeding monitoring; Table S15: Depth/compositional sensitivity and complete KEGG Level 3 and CAZy audits; Table S16: Bioinformatics software, databases, and parameters.

## Abbreviations

The following abbreviations are used in this manuscript:

ALB	Asian longhorned beetle
AA	Auxiliary activity
BH-FDR	Benjamini–Hochberg false discovery rate
CAZy	Carbohydrate-active enzymes database
CBM	Carbohydrate-binding module
CE	Carbohydrate esterase
FDR	False discovery rate
GH	Glycoside hydrolase
KEGG	Kyoto Encyclopedia of Genes and Genomes
KO	KEGG Orthology
LCA	Lowest common ancestor
LDA	Linear discriminant analysis
LEfSe	Linear discriminant analysis effect size
NR	Non-redundant protein database
ORF	Open reading frame
PCoA	Principal coordinates analysis
PERMANOVA	Permutational multivariate analysis of variance
PERMDISP	Permutational analysis of multivariate dispersions
PL	Polysaccharide lyase
TPM	Transcripts per million
