# Supplementary material for: Short-Term Feeding on Ecologically Distinct Dietary Plants Is Associated with Gut-Sample Bacterial and Archaeal Profiles in Adult Anoplophora glabripennis
Source: Insects. 2026 Jul 23;17(8):756. doi: 10.3390/insects17080756 (PMC13513481; doi:10.3390/insects17080756)
Supplement: Supplementary file 1 [file insects-17-00756-s001.zip › Supplementary Figures.pdf]

Supplementary Figures

Manuscript ID: insects-4412309

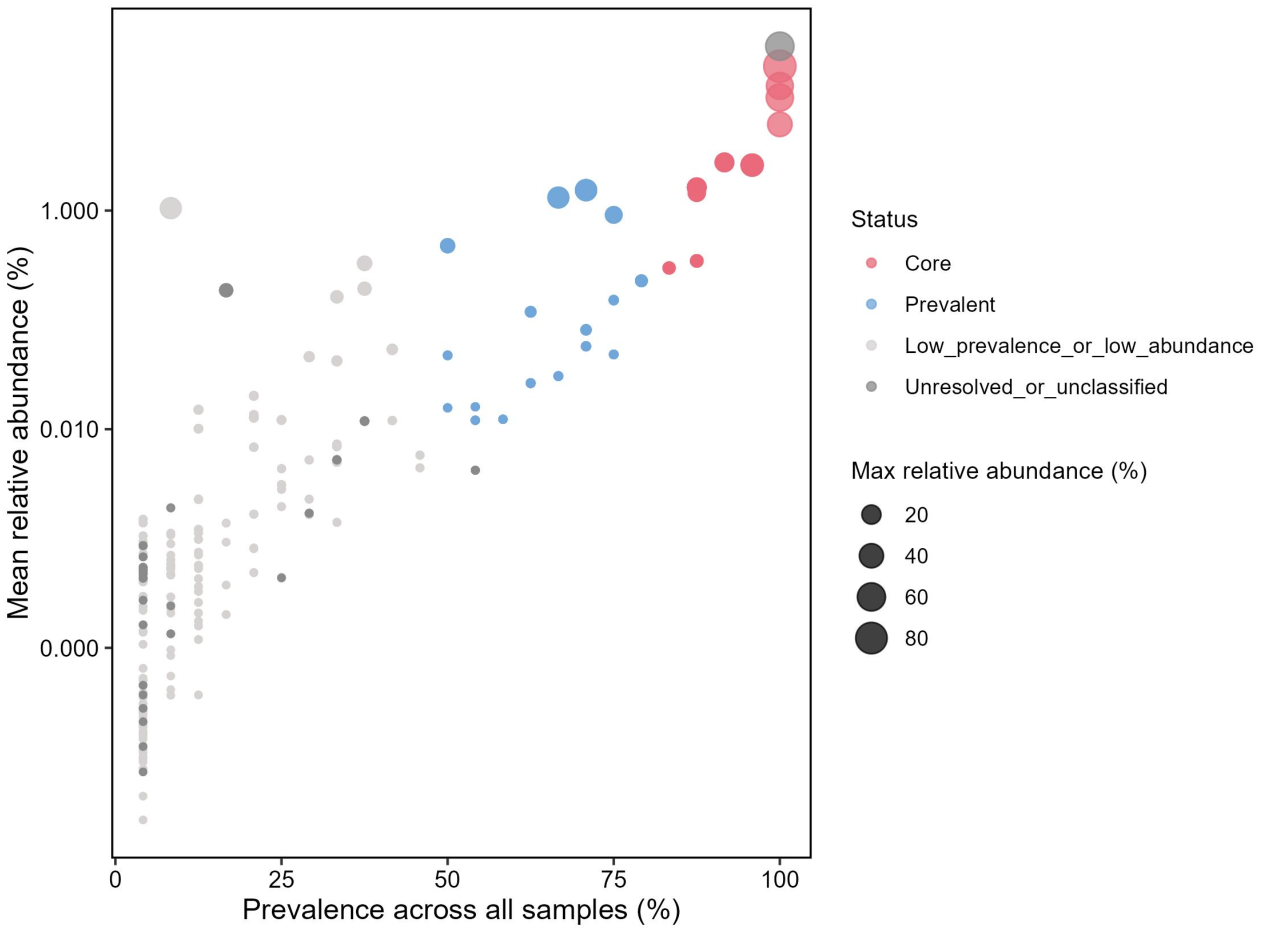

Figure S1. Prevalence and relative abundance of named genus-level bacterial and archaeal annotations across 24 adult Anoplophora glabripennis gut samples. Each point represents a named genus-level annotation. The x-axis shows prevalence across samples, the y-axis shows mean relative abundance, and point size indicates the maximum relative abundance observed among samples. Named genera detected in ≥80% of samples with a mean relative abundance ≥0.10% were operationally classified as core genera, whereas those detected in ≥50% of samples with a mean relative abundance ≥0.01% were classified as prevalent genera. Unresolved or ambiguous taxonomic assignments were retained in the complete taxonomic record but excluded from the classification and interpretation of named core and prevalent genera. These categories indicate repeated detection under the study-specific thresholds and do not establish stable gut residency.

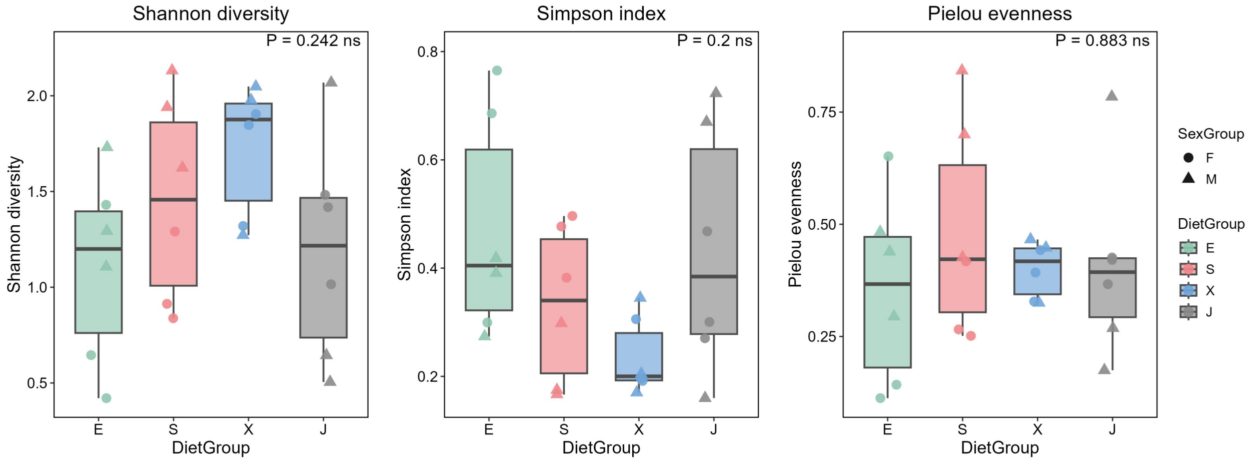

Figure S2. Alpha-diversity indices of bacterial and archaeal genus-level annotation profiles across DietGroups. Shannon diversity, Simpson diversity, and Pielou evenness were compared among DietGroups E, S, X, and J. Points represent individual samples, colors indicate DietGroup, and shapes indicate SexGroup. Global differences among DietGroups were evaluated using the Kruskal−Wallis test. None of the three alpha-diversity indices differed significantly among DietGroups.

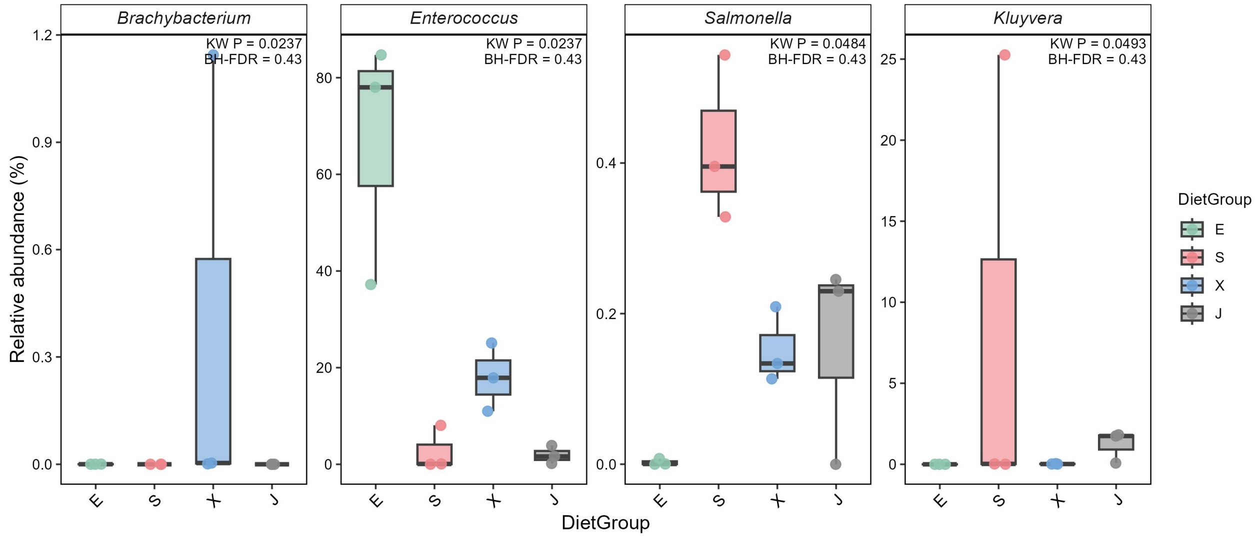

Figure S3. Exploratory female-only comparisons of candidate genus-level annotations among DietGroups. Boxplots show the relative abundances of four genus-level annotations with nominal DietGroup-associated differences in the female-only Kruskal−Wallis analysis. The displayed P values are unadjusted Kruskal−Wallis P values. None of the candidates remained significant after Benjamini−Hochberg correction, and they should not be interpreted as validated dietary biomarkers.

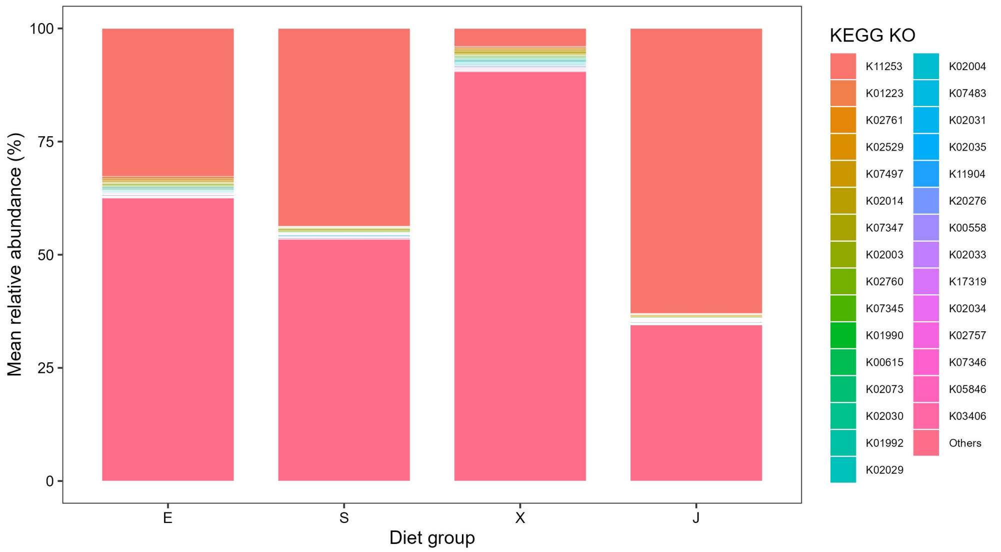

Figure S4. Relative-abundance composition of the top 30 KEGG Orthology annotations across DietGroups. Stacked bars show the mean relative-abundance composition of the top 30 KEGG Orthology (KO) annotations in DietGroups E, S, X, and J. KO profiles were generated from the retained bacterial/archaeal gene set and summarized at the DietGroup level. The figure provides a descriptive overview of the dominant KO annotations and complements the KO-level beta-diversity analysis. These annotation profiles do not demonstrate gene expression or functional activity.

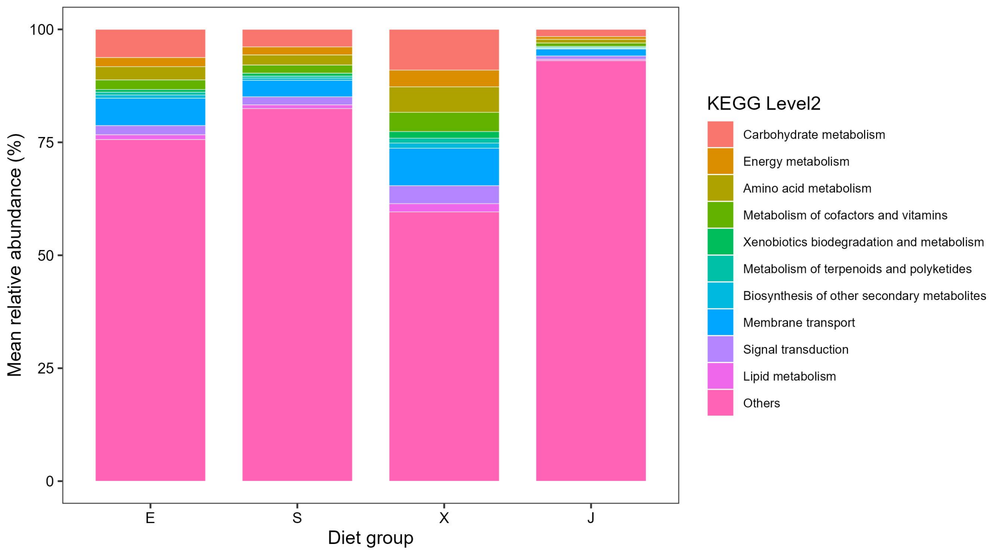

Figure S5. Relative-abundance composition of selected KEGG Level 2 functional categories across DietGroups. Stacked bars show the mean relative-abundance composition of selected KEGG Level 2 categories in DietGroups E, S, X, and J. Values were derived from KEGG annotations in the retained bacterial/archaeal gene set and provide a broad descriptive overview complementary to the complete and post hoc KEGG Level 3 analyses. The profiles represent annotation-based functional repertoire and should not be interpreted as evidence of gene expression or metabolic activity.

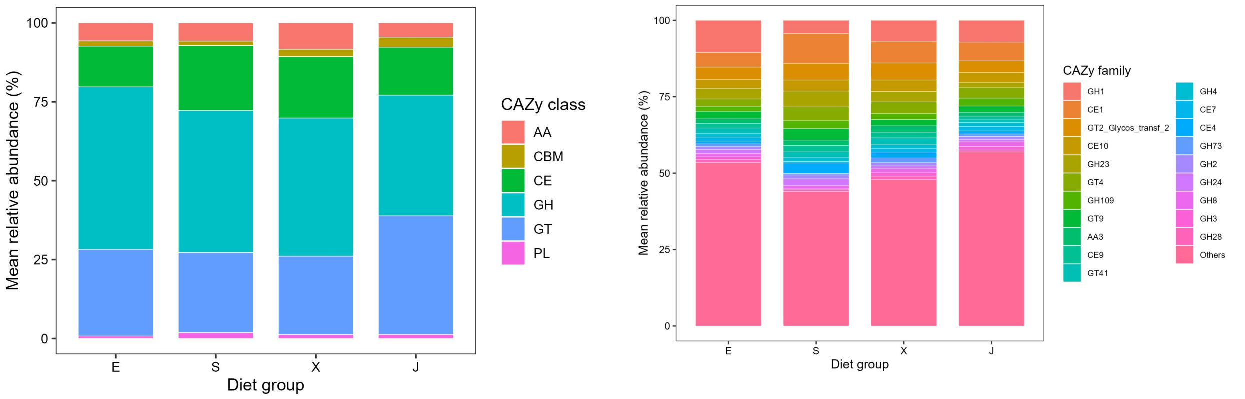

(A) **(B)**

Figure S6. CAZy class- and family-level annotation profiles in the retained bacterial/archaeal gene set. (A) Mean relative-abundance composition of the major CAZy classes across DietGroups, including glycoside hydrolases (GHs), carbohydrate esterases (CEs), auxiliary activities (AAs), carbohydrate-binding modules (CBMs), polysaccharide lyases (PLs), and glycosyltransferases (GTs). (B) Mean relative-abundance composition of the top 20 CAZy families across DietGroups; the remaining families are grouped as "Others." The CAZy profiles are descriptive because the family-level DietGroup PERMANOVA was not significant. They represent annotated carbohydrate-active enzyme repertoires rather than enzyme expression, enzyme activity, or diet-induced functional change.

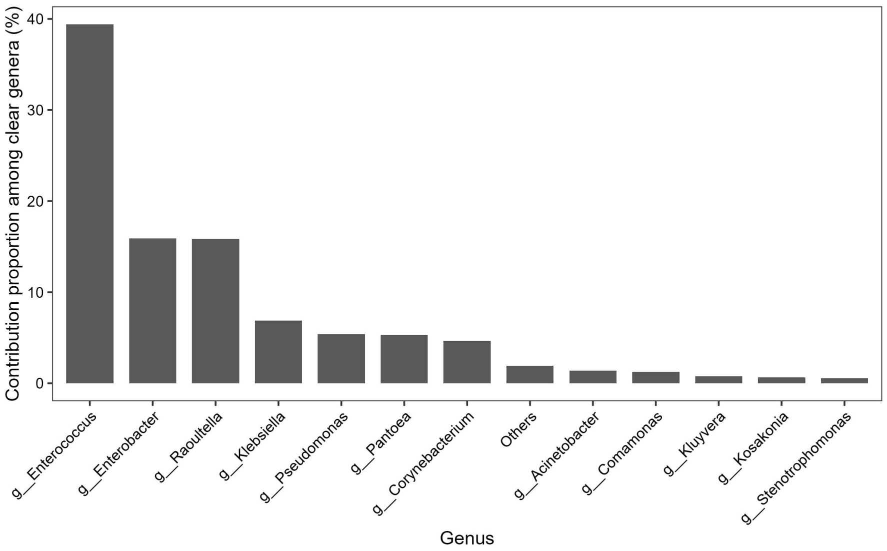

Figure S7. Total inferred annotation-based contribution of named genus annotations to 18 post hoc KEGG Level 3 pathways. The bar plot shows the total inferred contribution of each named genus annotation, summarized across the 18 pathways and all samples. Contributions were calculated by linking genus-level and KEGG pathway annotations through the same retained bacterial/archaeal gene records and summing the associated TPM abundance. Major inferred contributors included Enterococcus, Enterobacter, Raoultella, Klebsiella, Pseudomonas, Pantoea, and Corynebacterium. These estimates represent annotation-linked patterns and do not provide direct evidence of gene expression, in vivo functional activity, or a causal functional role for any genus.

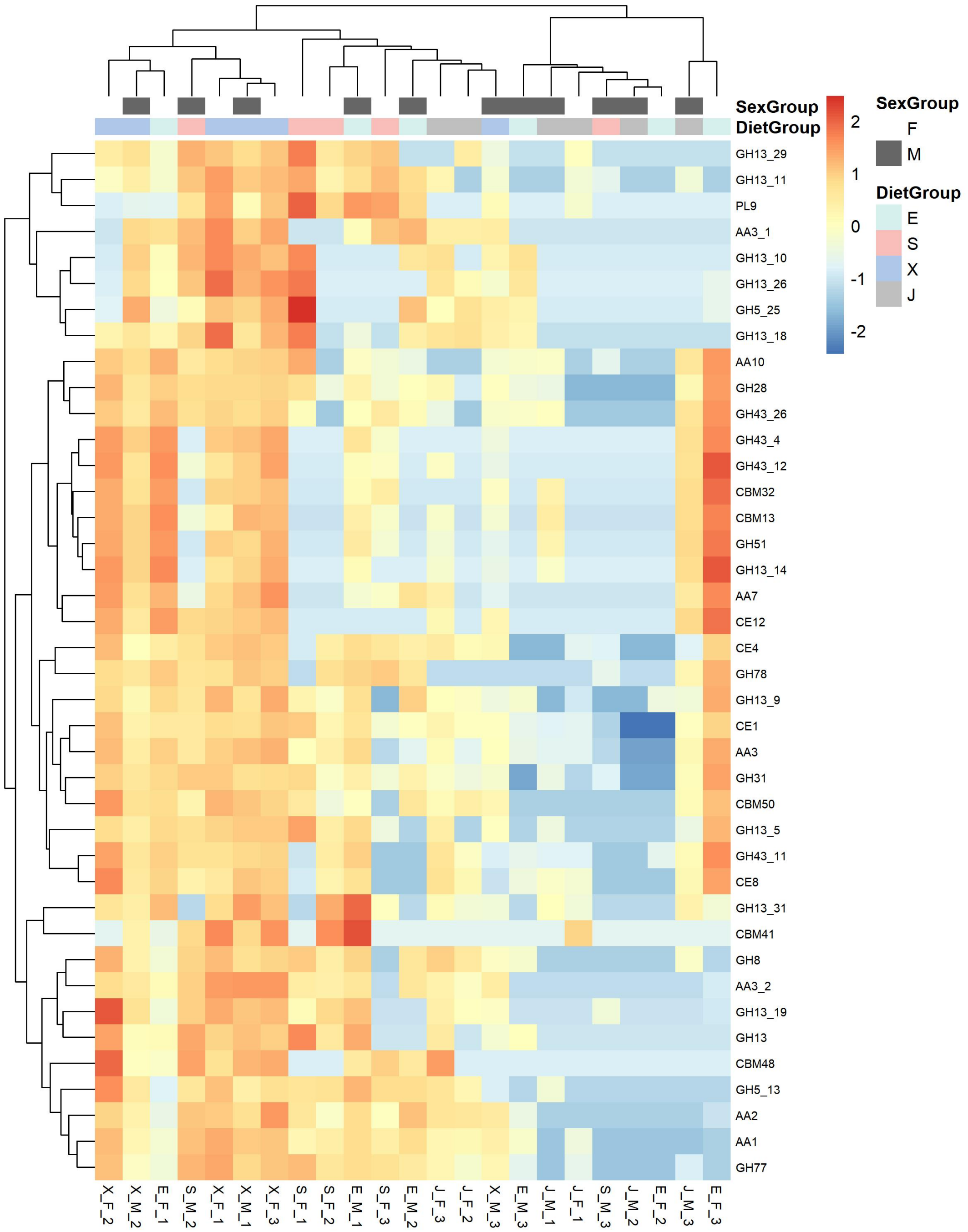

Figure S8. Descriptive heatmap of plant-cell-wall-related CAZy family abundance patterns across samples. Rows represent selected CAZy families associated with plant-polysaccharide processing, and columns represent individual gut samples. The annotation bars indicate DietGroup and SexGroup (F, female; M, male). TPM abundance was standardized separately for each CAZy family across the 23 displayed samples using a row-wise Z-score, calculated as $z = (x - \overline{x})/s$, where x is the family TPM abundance in a sample, $\overline{x}$ is the across-sample mean for that family, and s is its across-sample standard deviation. Colors therefore indicate relative within-family deviations from the family mean and should not be used to compare absolute abundance among different families. Rows and columns are hierarchically clustered according to the displayed standardized profiles. Sample S_M_1 is absent because no genes retained after bacterial/archaeal filtering had CAZy annotations. The heatmap is descriptive and does not indicate statistically supported diet effects, gene expression, or enzyme activity.

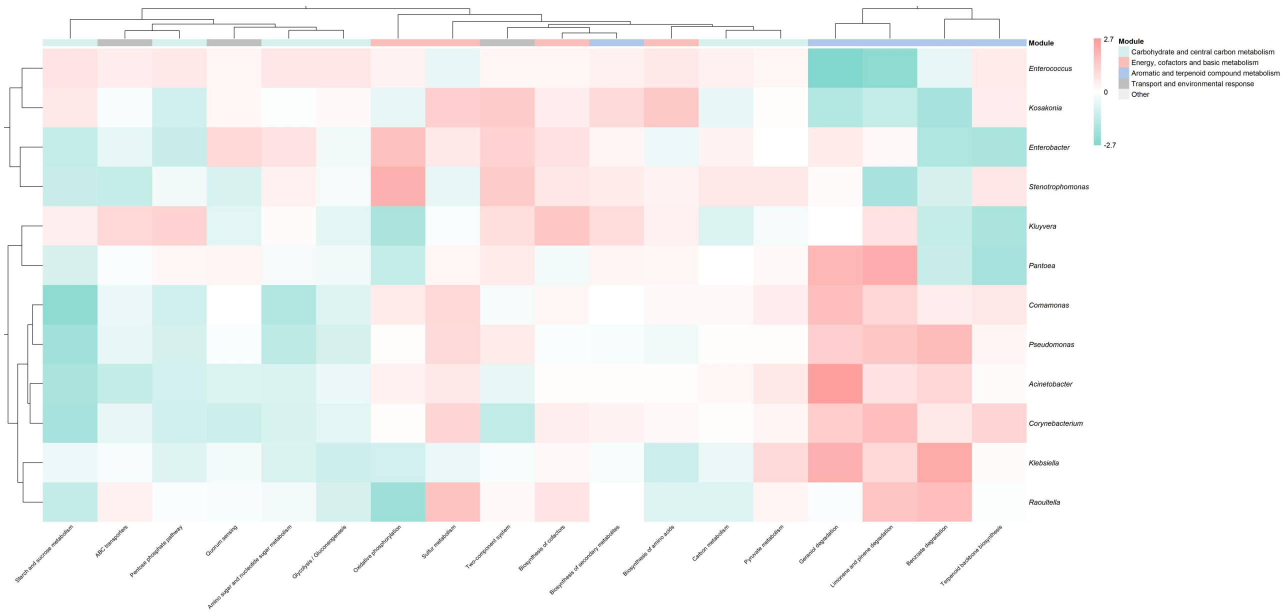

Figure S9. Detailed Genus × KEGG Level 3 inferred annotation-based contribution heatmap. Rows represent 12 displayed named genus-level annotations, and columns represent the 18 post hoc KEGG Level 3 pathways. The annotation strip above the columns indicates the broader functional module assigned to each pathway. Contribution values were calculated by linking genus and pathway annotations through the same retained bacterial/archaeal gene records and summarizing the associated TPM abundance across samples. Values were standardized separately for each genus across the 18 pathways using row-wise Z-scores; colors therefore show relative pathway-contribution patterns within each genus and should not be used to compare absolute contribution magnitudes among genera. Rows and columns are hierarchically clustered according to the displayed standardized profiles. The matrix is annotation-based and does not constitute MAG- or binning-based functional assignment, gene-expression or enzyme-activity evidence, correlation evidence, or proof of a direct functional role for any genus.
